# Supplementary material for: Dynamic oxygen adsorption on single-atomic Ruthenium catalyst with high performance for acidic oxygen evolution reaction
Source: Nat Commun. 2019 Oct 24;10:4849. doi: 10.1038/s41467-019-12886-z (PMC6813412; doi:10.1038/s41467-019-12886-z)
Supplement: Supplementary file 1 — Supplementary Materials [file 41467_2019_12886_MOESM1_ESM.pdf]

Supplementary Information

Dynamic oxygen adsorption on single-atomic Ruthenium catalyst with high performance for acidic oxygen evolution reaction

Cao et al.

Supplementary Note 1: Calculation of Roughness Factor (RF)

The roughness factor (RF) is calculated via taking the estimated ECSA and dividing by the geometric area of the electrode (A_g = 0.07 cm^2), and according to the (1):

$$RF=ESCA/A_g \quad (1)$$

Another activity metric sometimes reported in the electrocatalysis literature is the specific activity at a given overpotential. The definition of specific activity refer to the specific current density per ECSA (j_s), which is calculated by dividing the current density per geometric area (j_g) by the RF at a given overpotential and as shown in (2).

$$j_s=j_g/RF \quad (2)$$

Supplementary Note 2: Calculation for Turnover frequency (TOF)

At first, assuming each ruthenium atoms in the catalyst formed one active center. The numbers of Ru atoms number in Ru-N-C catalyst were calculated from the Ru molar mass and the mass loading ($m_{Loading}$) on the glass carbon electrode, which derived from the ICP results. The Ru content of catalyst revealed by ICP-AES measurement was ca. 1%, and the mass loading is about 0.280 mg cm^{-2}. Thus, the TOF the Ru-N-C electrocatalyst was calculated according to the equation (3).

$$TOF(O_2\ s^{-1})=\frac{\text{Total } O_2 \text{ turnovers per geometric area}}{\text{Active sites per geometric area}} \quad (3)$$

The number of total O_2 turnovers per geometric area was calculated from the current density (J_g) for the OER-LSV polarization:

$$=(|J|\frac{mA}{cm^2})(\frac{1C/s}{1000mA})(\frac{1mol}{96453.8C})(\frac{1mol}{4e})(\frac{6.023\times10^{23}}{1molO_2})$$

$$=1.56\times10^{15}\frac{O_2/s}{cm^2}\text{per}\frac{mA}{cm^2} \quad (4)$$

The upper limit of active sites density for Ru-N-C:

$$=\frac{0.280mg\ cm^{-2}\times10^{-3}\times1\%\times6.023\times10^{23}}{101.07g\ mol^{-1}}\text{per cm}^2 \quad (5)$$

$$=1.67\times10^{23}\ \text{Ru site per cm}^2$$

Finally, the TOF of Ru-N-C can be obtained via the current density from the OER-

LSV polarization curves and according to (6):

$$TOF_{Ru\text{-}N\text{-}C} = \frac{1.56\times10^{15}}{1.67\times10^{16}}\left|J\right| = 0.093\left|J\right|. \tag{6}$$

Particularly, at the overpotential of 267 and 300 mV, the current density of OER are 10 and 40 mA cm^{-2}, respectively. Therefore, the TOF values of Ru-N-C electrocatalyst were calculated to be:

$$TOF_{(267mV)} = 0.093\times10=0.93\ O_2\ s^{-1} =3348\ O_2\ h^{-1}. \tag{7}$$

$$TOF_{(300mV)} = 0.093\times40=3.72\ O_2\ s^{-1} =13392\ O_2\ h^{-1}. \tag{8}$$

Similarly, the upper limit of active sites density for 20 *wt*% RuO_2/C:

$$=2.53\times10^{17}\ \text{Ru site per cm}^2. \tag{9}$$

So, the TOF values of RuO_2/C electrocatalyst were calculated to be:

$$TOF_{OER} = \frac{1.56\times10^{15}}{2.56\times10^{17}}\left|J\right| = 0.006\left|J\right|. \tag{10}$$

Particularly, the current density of OER for RuO_2/C are 3.25 and 9.09 mA cm^{-2} at the overpotential of 267 and 300 mV, respectively. Therefore, the TOF values of RuO_2 electrocatalyst were calculated to be:

$$TOF_{(267mV)} = 0.006\times3.25=0.0195\ O_2\ s^{-1} =8.5\ O_2\ h^{-1}. \tag{11}$$

$$TOF_{(300mV)} = 0.006\times9.09=0.054\ O_2\ s^{-1} =196.34\ O_2\ h^{-1}. \tag{12}$$

Supplementary Note 3: Mass activity calculation

Mass activity (A g_{metal}^{-1}) was derived from the current density (mA cm^{-2}) that normalized by the mass loading (0.280 mg cm^{-2}) and per metal at a certain applied overpotential. As the following equation exhibited the mass activity of Ru-N-C:

$$\text{Mass activity}_{Ru\text{-}N\text{-}C}=\frac{|J|}{0.280\times1\%}=357.1\ |J|\ A\ g_{metal}^{-1}\ \text{per cm}^2. \tag{13}$$

The current density for Ru-N-C are 10 and 40 mA cm^{-2} at the overpotential of 267 and 300 mV, respectively. Therefore, the mass activity were calculated to be:

$$\text{Mass activity}_{267mV} = 357.1\times10 = 3571\ A\ g_{metal}^{-1}. \tag{14}$$

$$\text{Mass activity}_{300mV} = 357.1\times40 = 14284\ A\ g_{metal}^{-1}. \tag{15}$$

Similarly, the mass activity of RuO_2/C were calculated to be:

$$\text{Mass activity}_{RuO_2} = \frac{|J| \times 133.07\text{g/mol}}{0.280 \times 20\% \times 101.07\text{g/mol}} = 23.5\ |J|\ \text{A}\ g_{metal}^{-1}\ \text{per cm}^2. \quad (16)$$

Particularly, the current density for RuO_2/C are 3.25 and 9.09 mA cm^{-2} at the overpotential of 267 and 300 mV, respectively. Therefore, the mass activity can be obtained according to (17) and (18) :

$$\text{Mass activity}_{267mV} = 23.5 \times 3.25 = 76.4\ \text{A}\ g_{metal}^{-1}. \quad (17)$$

$$\text{Mass activity}_{300mV} = 23.5 \times 9.09 = 213.6\ \text{A}\ g_{metal}^{-1}. \quad (18)$$

Supplementary Note 4: Computational methods

The theoretical overpotential of OER was calculated by using the computational hydrogen electrode model, i.e., at the standard conditions, pH=0, $H^+ + e^- \rightarrow 1/2H_2$, $G_{H++e-} = 1/2G_{H2} - eU$, U is the electrode potential and e is unit charge, respectively.[1] The free energy profile calculations were computed via the following formula:

$$G = E + ZPE - TSS \quad (19)$$

ZPE is the zero-point vibration energy, T is the room temperature (298.15K) and S is the entropy.

$$ZPE = \frac{1}{2} h\nu \quad (20)$$

$$S_{vib} = R\left\{ \frac{h\nu}{k_B T}\left[\exp(\frac{h\nu}{k_B T}) - 1 \right]^{-1} - \ln\left[1 - \exp(-\frac{h\nu}{k_B T}) \right] \right\} \quad (21)$$

Where ν is the vibrational frequency of adsorbed intermediates and can be calculated using a finite difference method, R is gas constant, h is Planck’s constant and k_B is Boltzmann’s constant. During the calculation of vibrational frequency, only the adsorbed intermediates were allowed to relax while the rest of atoms in the system were fixed.

The OER mechanism was considered following the four elementary steps, and each of step consists of a single proton electron transfer:

$$2H_2O + * \rightarrow OH^* + H_2O + e^- + H^+$$
$$OH^* + H_2O \rightarrow O^* + H_2O + e^- + H^+$$
$$O^* + H_2O \rightarrow OOH^* + e^- + H^+$$
$$OOH^* \rightarrow O_2 + e^- + H^+$$

$$\Delta G_1 = \Delta G_{OH} + \frac{1}{2}\Delta G_{H_2} - eU$$
$$\Delta G_2 = \Delta G_O - \Delta G_{OH} + \frac{1}{2}\Delta G_{H_2} - eU$$
$$\Delta G_3 = \Delta G_{OOH} - \Delta G_O + \frac{1}{2}\Delta G_{H_2} - eU$$
$$\Delta G_4 = 4.92\left[eV\right] - \Delta G_{OOH} + \frac{1}{2}\Delta G_{H_2} - eU$$

The theoretical overpotential η^{OER} at standard conditions can be written as:

$$\eta^{\mathrm{OER}} = \frac{\max\left[\Delta G_1, \Delta G_2, \Delta G_3, \Delta G_4\right]}{e} - 1.23\ \mathrm{V} \qquad (22\ )$$

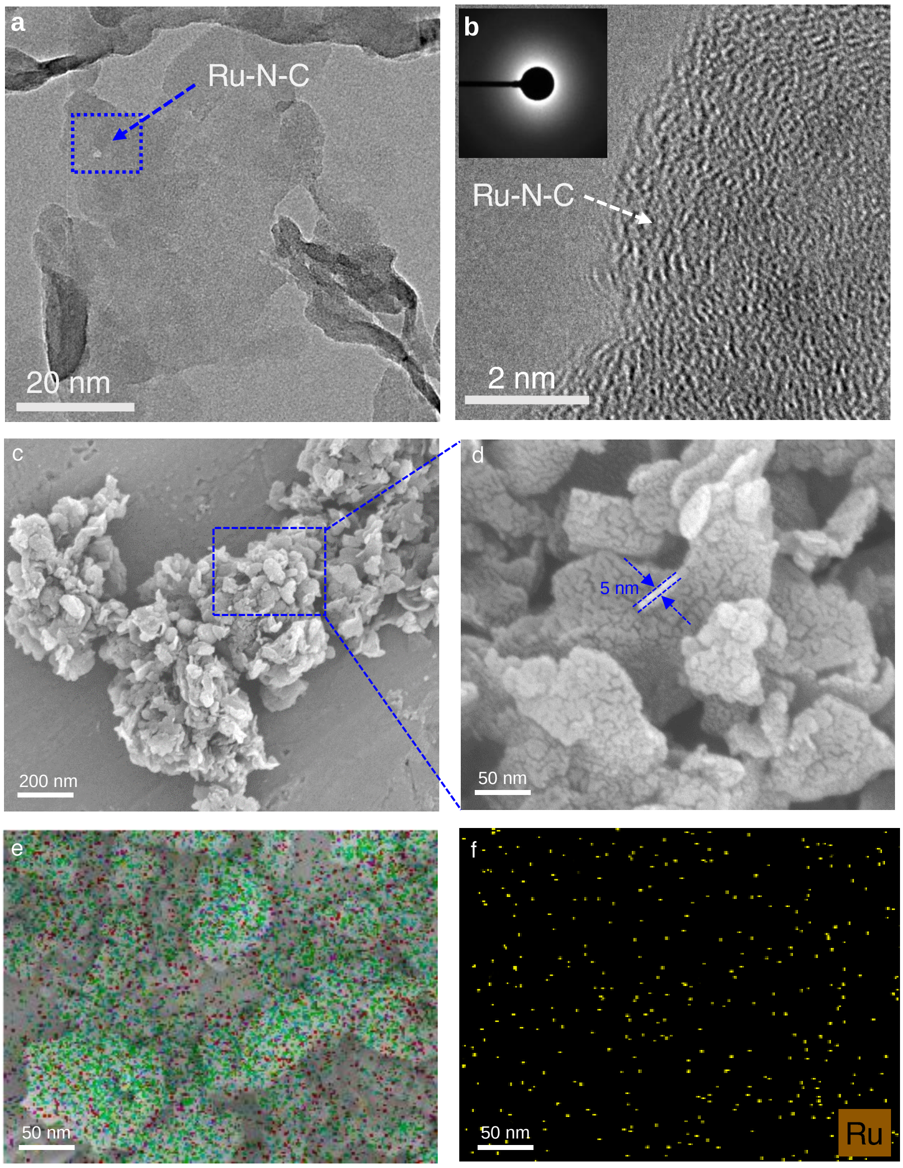

Supplementary Figure 1. The morphology of Ru-N-C. (a) Low magnification and (b) Low magnification TEM images of Ru-N-C catalyst. Insert: the corresponding SAED pattern. (c) Low magnification and (d) high magnification SEM images of Ru-N-C. (e) and (f) SEM-EDS elemental mapping of Ru-N-C catalyst.

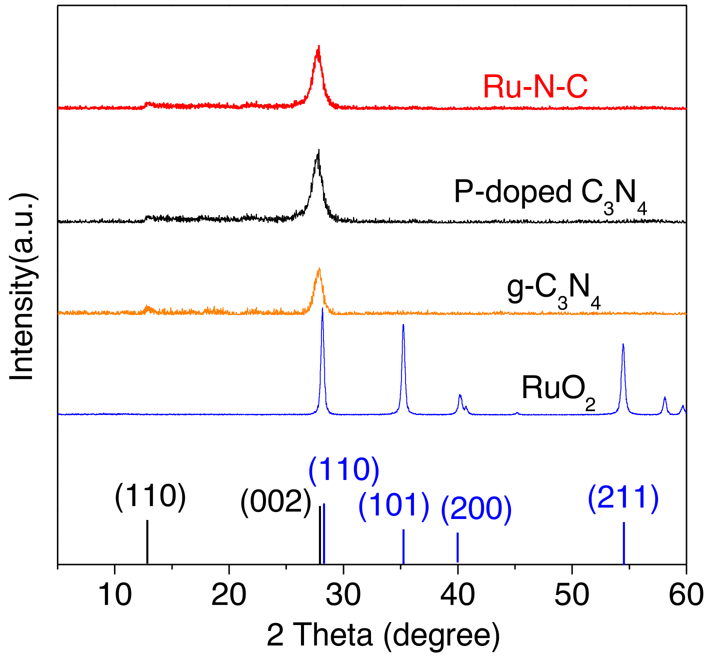

Supplementary Figure 2. XRD patterns for Ru-N-C and the references. A diffraction peak at ca. 27.4 °corresponds to the (002) interplanar distance of 0.3 nm for the melon network. Another peak at 13.0 ° can be assigned to (100) in-planar ordering of tri-s-triazine.[2] The diffraction peaks are well indexed to the (110), (101), (200) and (211) planes of tetragonal RuO_2 phase.[3]

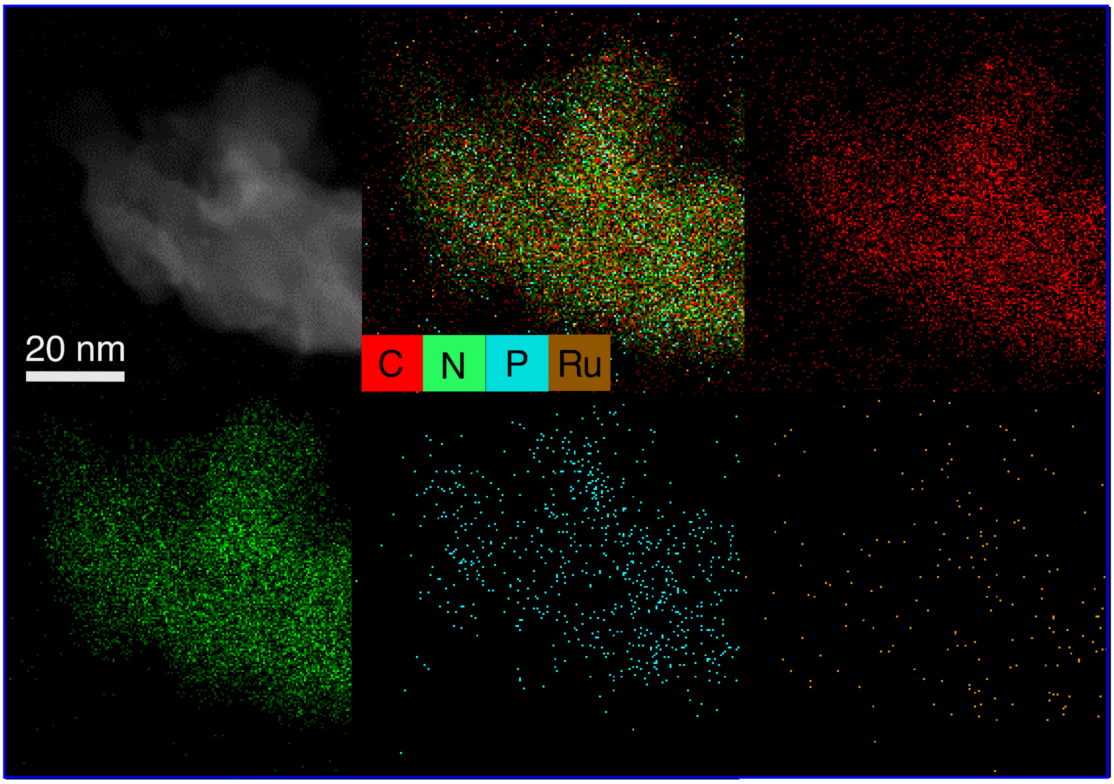

Supplementary Figure 3. STEM-EDS elemental mapping of Ru-N-C catalyst.

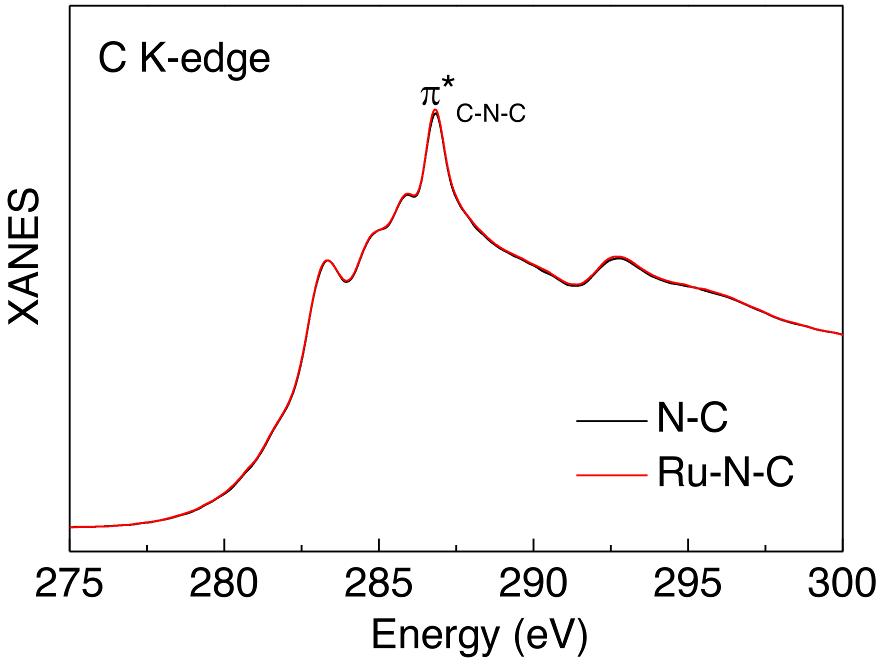

Supplementary Figure 4. C K-edge XANES spectra for Ru-N-C and pristine N-C.

The shape peak at around 288 eV is ascribed to the π* bond formed by the C-N rings.[4]

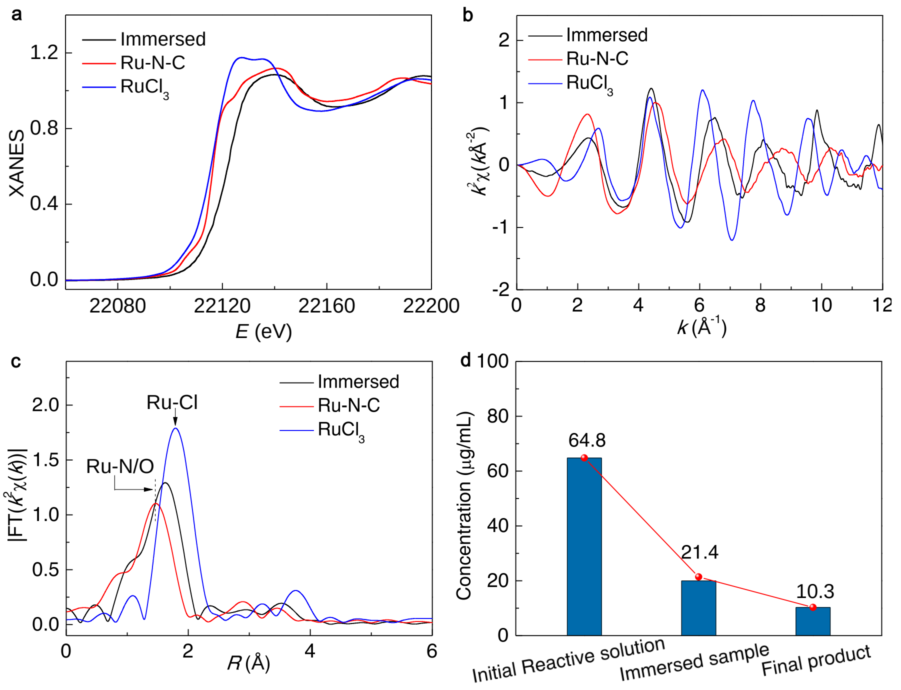

Supplementary Figure 5. Exclusion of the presence of Cl. (a) Ru K-edge XANES spectra for immersed Ru-N-C, Ru-N-C, and $RuCl_3$. (b) $k^2\chi(k)$ oscillations of Ru K-edge EXAFS oscillation functions and (c) the corresponding FT curves. (d) The contents of Cl species. The initial reactive solution represented the $RuCl_3$ aqueous solution mixed with the homogeneous C-N aqueous at 70 ℃ for 5 h, while the immersed sample is the dried mixture via rotary dryer.

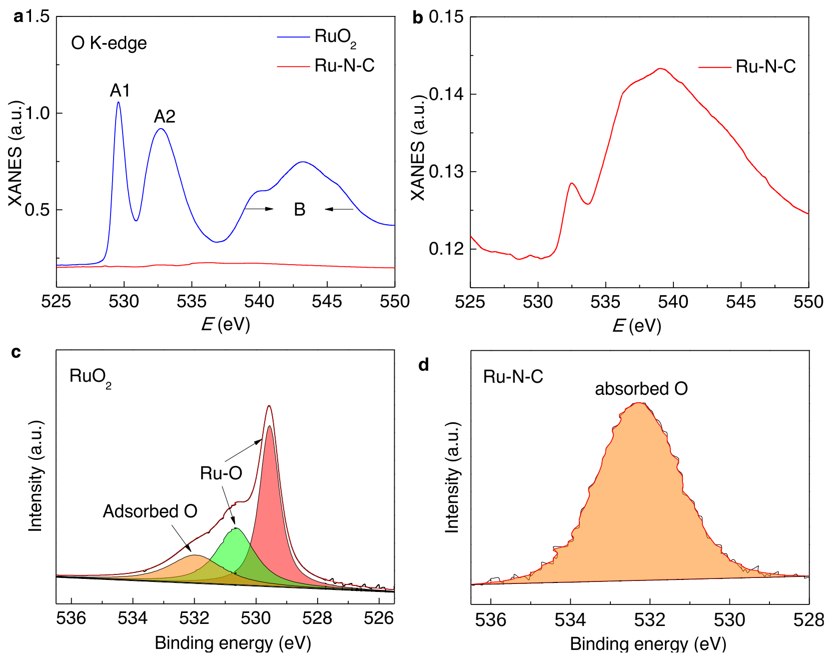

Supplementary Figure 6 Exclusion of the presence of Ru-O coordination. (a), (b) O K-edge XANES for RuO_2 and Ru-N-C. (c), (d) O 1*s* XPS high-resolution spectra for RuO_2 and Ru-N-C. The XANES spectrum of RuO_2 reference displays the diploe electron transitions from the core level O1*s* into unoccupied O2*p* projected states above the Fermi level. Due to hybridization between O 2*p* and Ru 4*p*, the XANES spectrum splits into double peaks A1 and A2 located at around 529 and 532 eV, respectively. Whereas the broad peak B, centered at ca. 544 eV, is related to the transitions into O2*p*-Ru4*sp* hybridized bands.[5] In contrast, the XANES signals for the Ru-N-C and N-C support are very weak, suggesting oxygen contents is quite low. Although the O K-edge XANES spectra of Ru-N-C and N-C XANES can be seen after magnification, their shapes are completely different from RuO_2. The metallic oxygen peaks at ca. 529 and 532 eV could be hardly discerned, ruling out the presence of Ru-O coordination in ex-situ Ru-N-C sample, which is consistent with the XAFS fitting results. To further exclude the possibility of the formation of Ru-O bonds, we also performed the O 1*s* XPS measurements on the Ru-N-C and RuO_2. The high-resolution O 1*s* XPS spectrum of RuO_2 shown in Supplementary Fig. 6c displays the dominant peaks at 529.6 and 530.7 eV, which are essentially assigned to the lattice oxygen. However, another weak signal was observed at ca. 532 eV corresponding to the adsorbed O_2.[6] Contrarily, only one dominant peak at around 532 eV ascribed to the adsorbed O_2 was detected in the O 1*s* XPS spectrum for Ru-N-C (Supplementary Fig. 6d), indicating the absence of Ru-O chemical bond in ex-situ Ru-N-C sample, in agreement with the results of O K-edge XANES. It is worth noting that the presence of adsorbed O_2 is unavoidable in the air, while it does not contribute to or influence the catalyst performance.

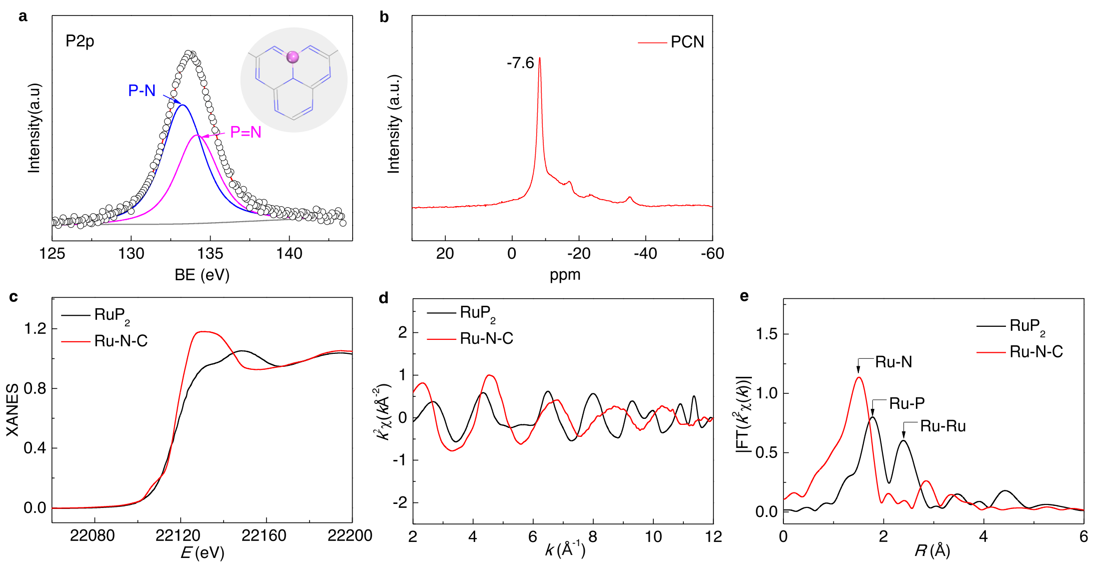

Supplementary Figure 7 The coordination of P-N. (a) The high-resolution P 2*p* XPS spectra for the Ru-N-C. Insert: the schematic model of P doping at C site. (b) ^{31}P solid-state NMR spectra of N-C. Insert: the schematic model of P doping at C site. (c) Ru K-edge XANES spectra for RuP_2, RuO_2 and Ru_1-N-C. (d) $k^2\chi(k)$ oscillations of Ru K-edge EXAFS oscillation functions and (e) the corresponding FT curves. The high-resolution XPS spectra of P 2p. Insert: the schematic structure of N-C and the pink ball represents P atom. At first, the P 2p XPS spectra possesses the peak at binding energy of 133.5 eV, corresponding to the coordination of P and N (P-O bonding would be ca. 1 eV higher while P-C bonding would be ca. 1-2 eV lower than P-N). The XPS peak can be deconvoluted into two contributions located at 133.0 eV and 134 eV, which are ascribed to the P-N and P=N coordination, respectively. Therefore, this result strongly indicates that P atoms most probably substitute C atoms in g-C_3N_4 to form P-N coordination.[7,8] More importantly, the main purpose of P doping is to improving electrical conductivity, which have been approved by our previous work.[9]

The XANES spectrum of the Ru-N-C is quite different from that of the RuP_2 reference. The FT curve of RuP_2 reference is characterized by two main peaks at 1.78 Å and 2.9 Å, corresponding to the nearest Ru-P and Ru-Ru coordination, respectively, which are strikingly different from those of the Ru-N-C sample.

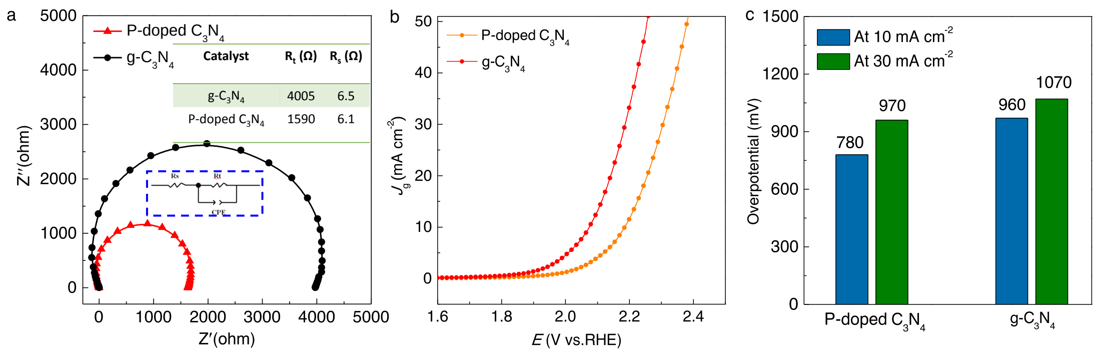

Supplementary Figure 8 The role of P doping. (a) EIS of the g-C_3N_4 and the P-doped C_3N_4. Insert: the equivalent circuit diagram and the corresponding impedance data. (b) Electrocatalytic OER performance of the P-doped C_3N_4 and g-C_3N_4 electrocatalysts in 0.5 M H_2SO_4 electrolyte. (c) Overpotential obtained from OER polarization curves at the current densities of 10 and 30 mA cm^{-2}.

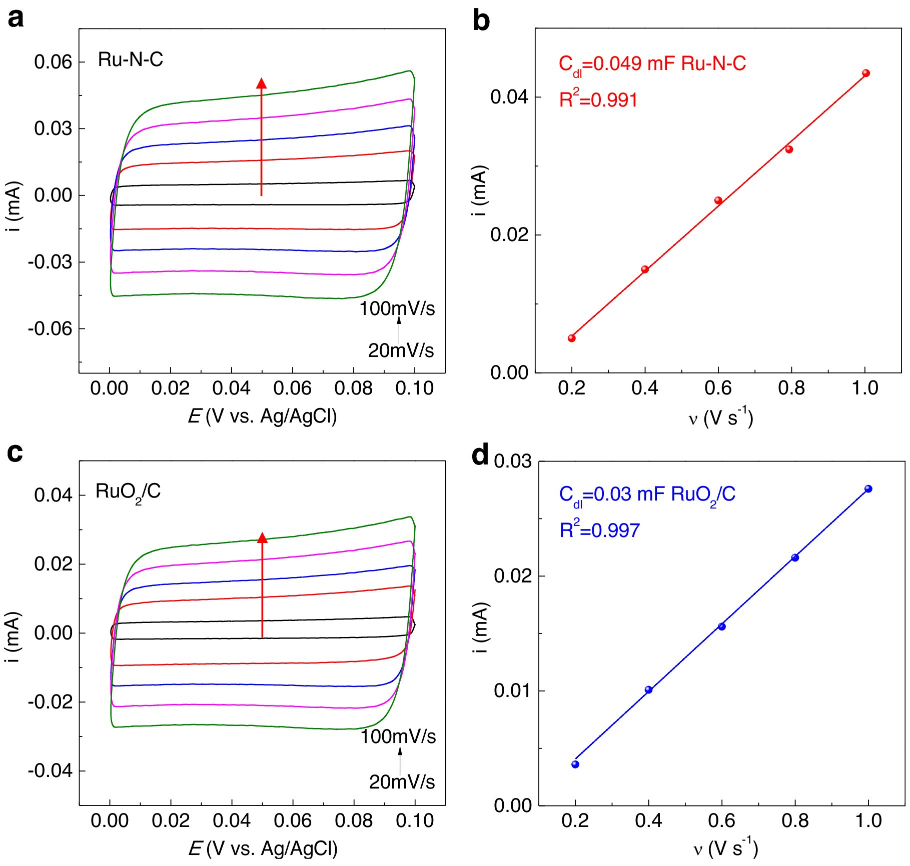

Supplementary Figure 9 Double-layer capacitance measurements. (a), (c) CVs were conducted in a non-Faradaic region of voltammogram at the following scan rate: 0.02 (black cycle), 0.04 (red cycle), 0.06 (blue cycle), 0.08 (pink cycle), 0.1 V s^{-1} (green cycle). The working electrode was held at each potential vertex for 20 s before beginning the next sweep and all current is assumed to be due to capacitive charging. (c), (d) The anodic charging currents measured at 0.05 V vs. RHE plotted as a function of scan rate. The C_{dl} are absolute value of the slope of the liner fits to the data.

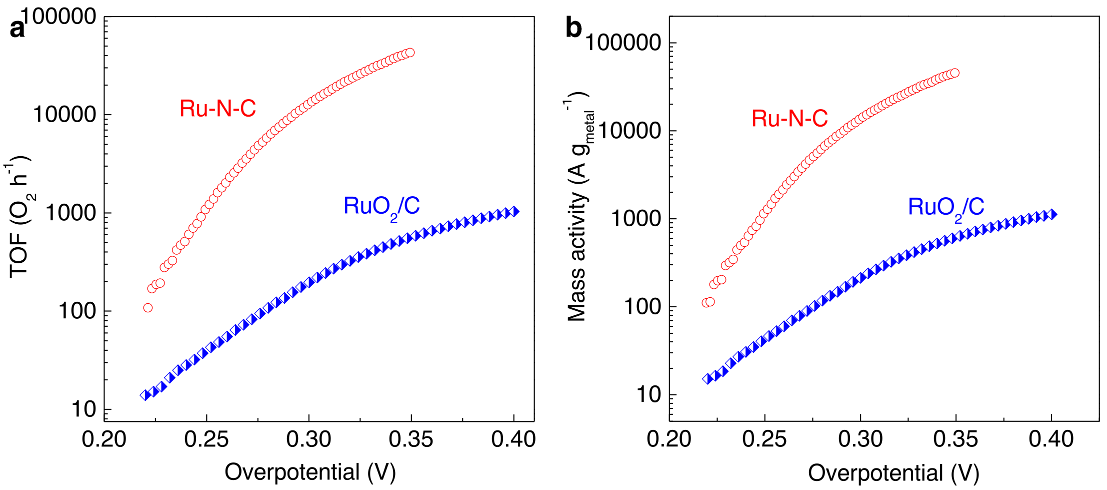

Supplementary Figure 10. The turnover frequencies. (a)TOF and (b) mass activity plots of the Ru-N-C and RuO_2/C.

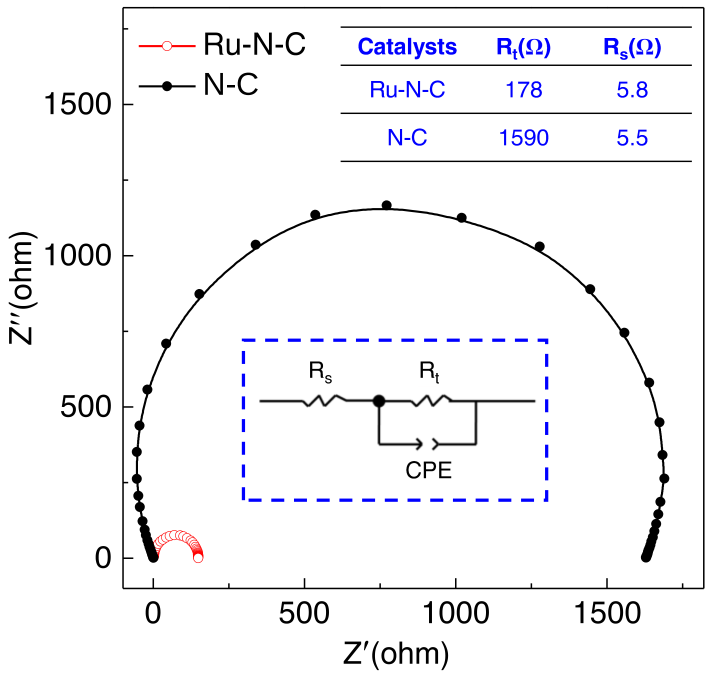

Supplementary Figure 11. Electrochemical impedance spectroscopy (EIS). Insert: the equivalent circuit diagram and the corresponding fitted parameters. It can be obviously found that the Ru-N-C exhibits the smaller diameter and R_t value in relative to that pristine N-C. This indicates that the enhanced charge transfer from the electrode to electrolyte, which is critical for electrocatalytic activity for OER.

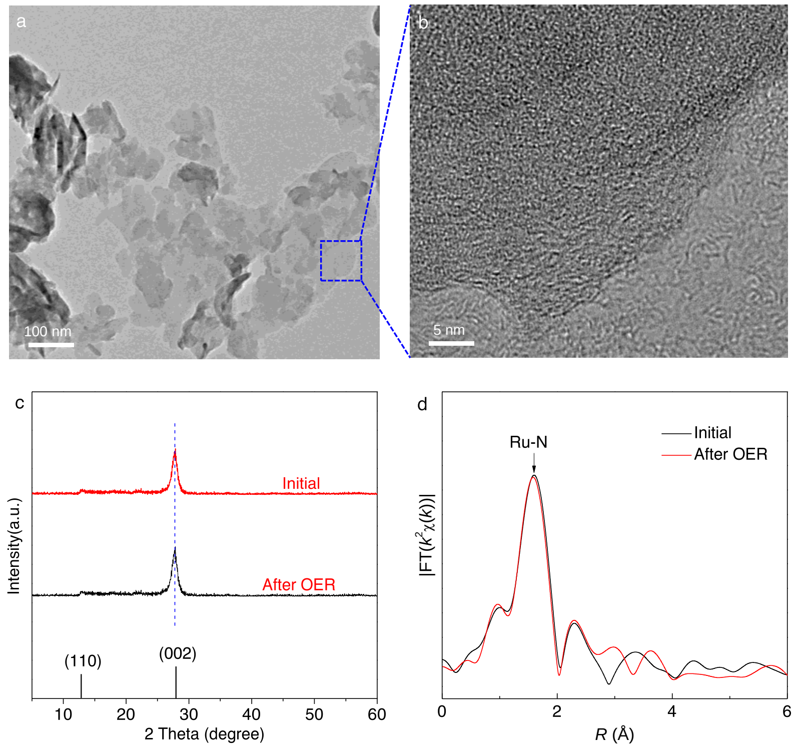

Supplementary Figure 12. The structural stability test. (a) Low-magnification, (b) high-magnification TEM images, and (c) XRD patterns for the Ru-N-C catalyst after long-time electrolysis. (d) The k^2-weighted Fourier transform EXAFS spectra of catalyst before and after electrochemical test. Ru-N-C catalyst substantially maintained layered structure with no nanoparticles can be found after a long-term electrolysis. Moreover, the XAFS and XRD results further demonstrated that the Ru in Ru-N-C catalyst remain the atomic dispersion without aggregation into the particles. These results suggest that the Ru-N-C can stably trigger the acid OER.

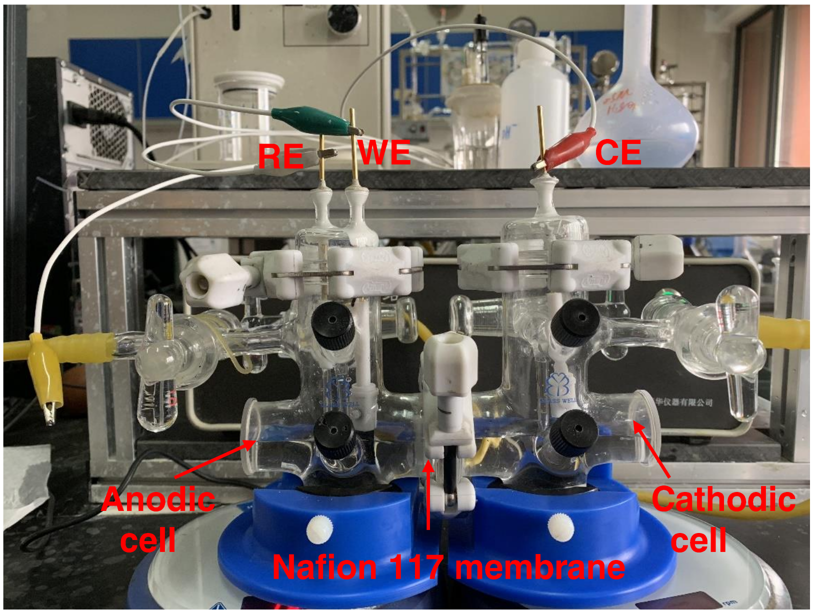

Supplementary Figure 13. Digital photograph of an H-type electrochemical cell. WE: working electrode, CE: counter electrode and RE: reference electrode.

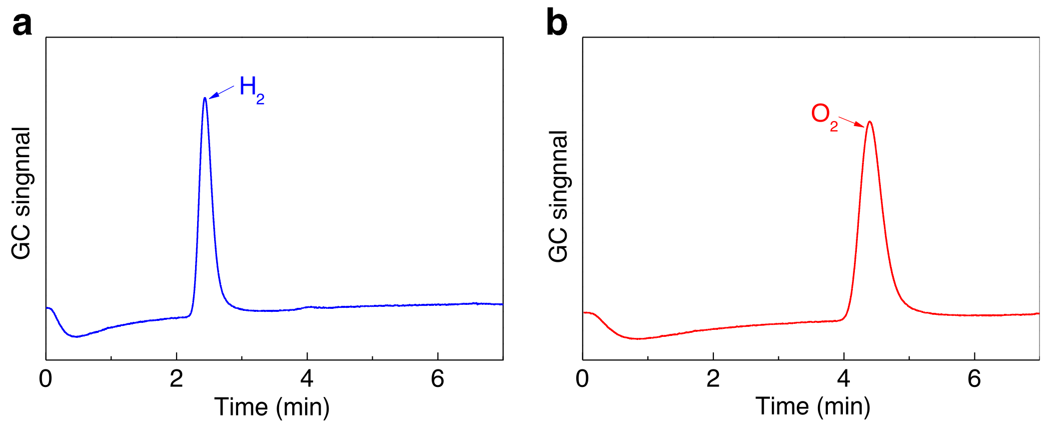

Supplementary Figure 14. Gas chromatography survey for Ru-N-C||Pt/C. (a) The GC signal of H_2 at cathodic cell and (b) for O_2 at anodic cell.

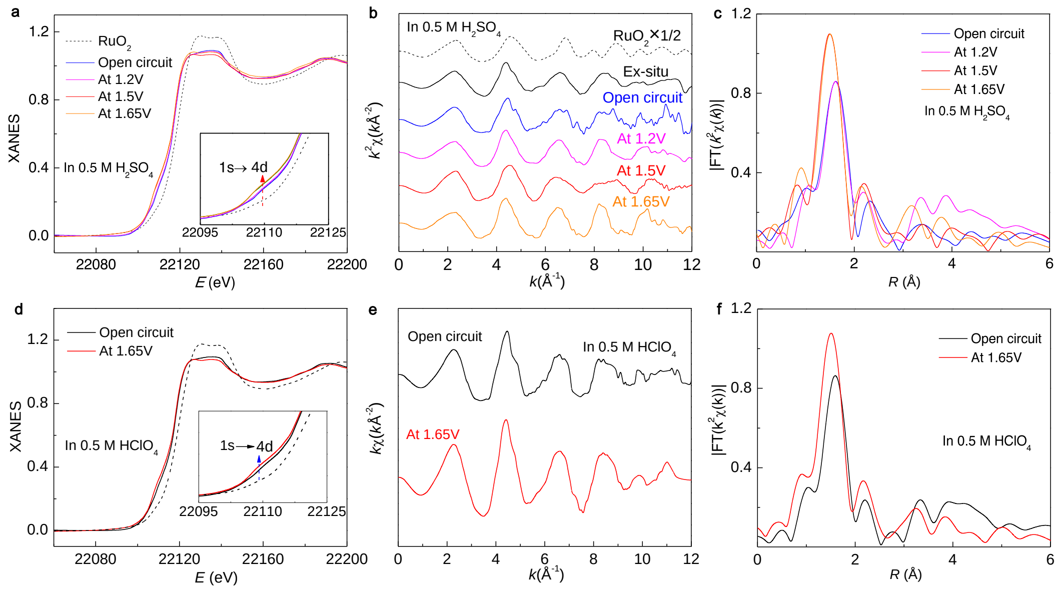

Supplementary Figure 15. The *Operando* XAFS results. (a) *Operando* XANES spectra for Ru-N-C at different applied voltages from open-circuit condition to 1.65 V vs. RHE during OER in H_2SO_4 electrolyte. The corresponding $k^2\chi(k)$ oscillations (b), and Fourier transforms (c) for (a). (d) The Ru K-edge XANES spectra for Ru-N-C at open-circuit condition and at 1.6 V in in 0.5 M $HClO_4$ electrolytes. The corresponding $k^2\chi(k)$ oscillations (b), and Fourier transforms (c) for (d). It can be found that the XANES and EXAFS spectra for the catalyst at 1.65 V in 0.5 M $HClO_4$ solution display the similar evolutions, as compared to those in H_2SO_4 electrolyte. This further confirm that the observed intermediate species is from the oxygen adsorption. All potentials are normalized to RHE.

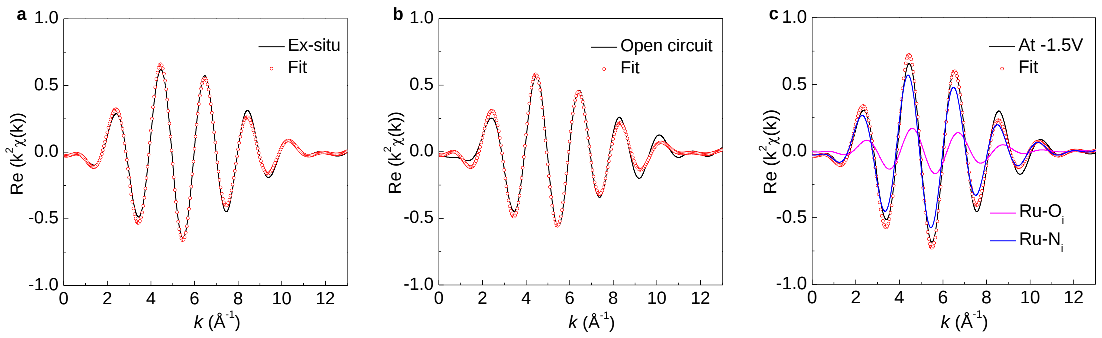

Supplementary Figure 16. First-shell fitting paths for the catalyst. (a) ex situ, (b) open circuit and (c) at1.5V vs. RHE.

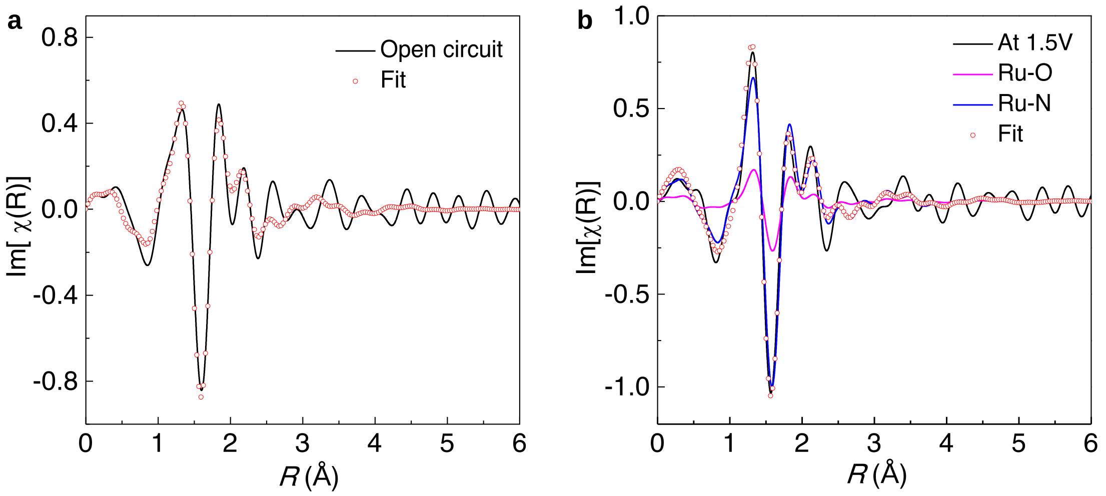

Supplementary Figure 17. The imaginary-part results of the EXAFS spectra. (a) Ru-N-C (a) under open circuit, and (b) at 1.5 V *vs*. RHE.

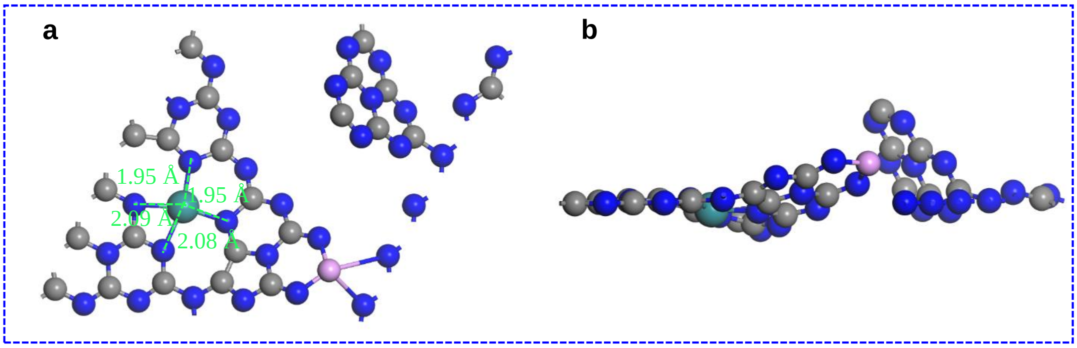

Supplementary Figure 18. The configurations for Ru-N-C. (a) Top and (b) side views of atomic configuration for ex-situ Ru-N-C single-site catalyst. The balls in grey, blue, and light green represent C, N and Ru atoms, respectively.

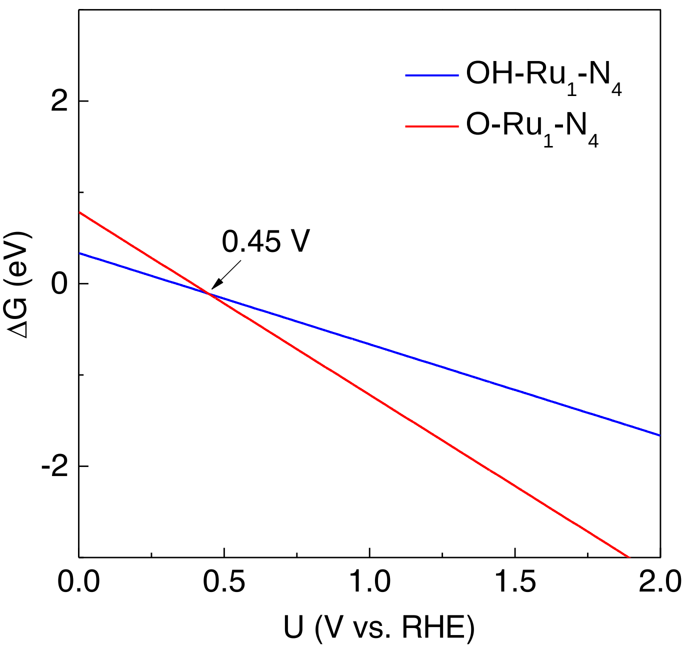

Supplementary Figure 19. The surface phase diagram of Ru_1-N_4 site as a function of applied potential (U).

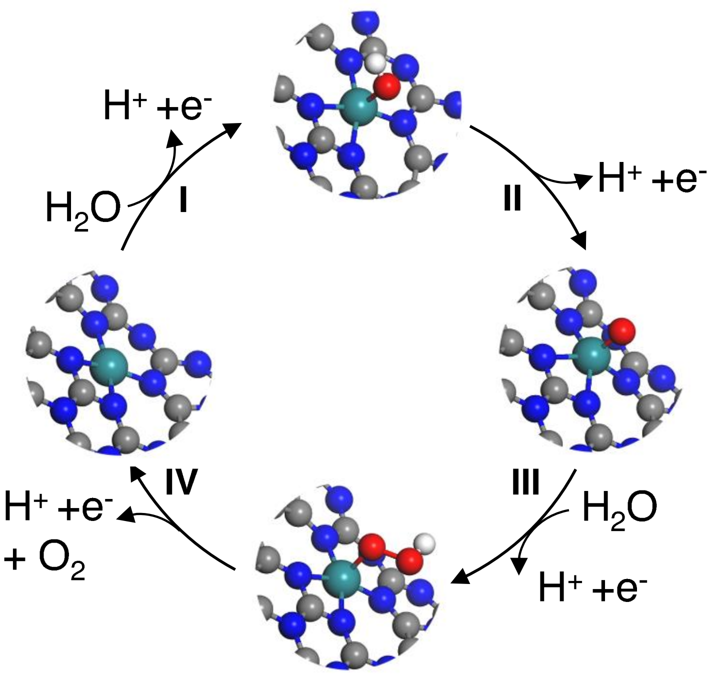

Supplementary Figure 20. The schematic of the whole OER mechanism on ex-situ Ru_1-N_4 in the acidic electrolyte. The balls in grey, blue, red, white, and light green represent C, N, O, H and Ru atoms, respectively.

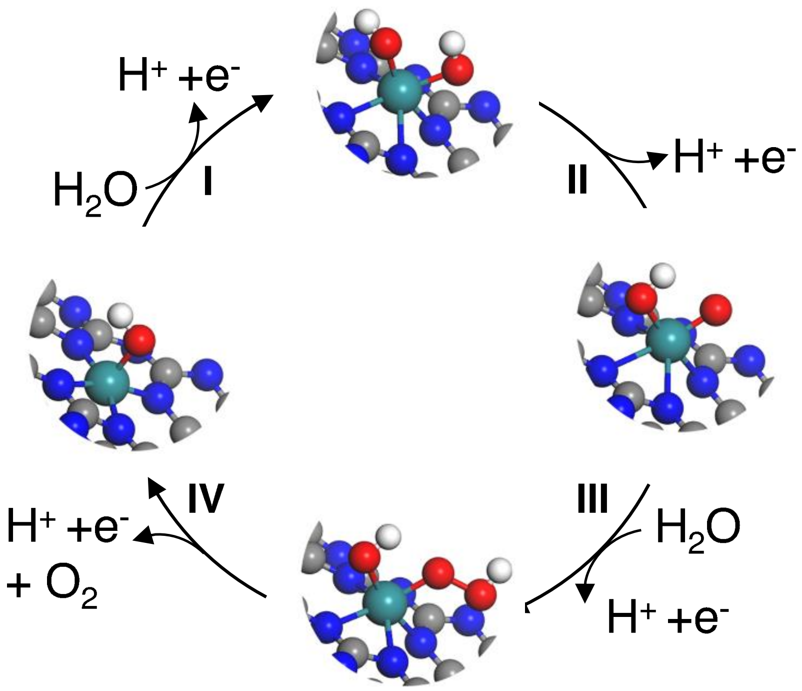

Supplementary Figure 21. The schematic of the whole OER mechanism on HO-Ru_1-N_4 in the acidic electrolyte. The balls in grey, blue, red, white, and light green represent C, N, O, H and Ru atoms, respectively.

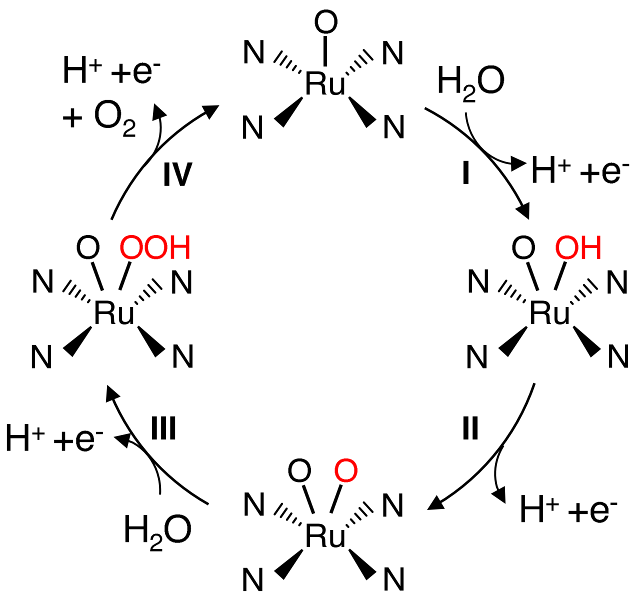

Supplementary Figure 22. The schematic of the whole OER mechanism on O-Ru$_1$-N$_4$ in the acidic electrolyte.

Supplementary Table 1. Structural parameters extracted from the quantitative EXAFS curve-fitting via ARTIMIS module of IFEFFIT. (The fixed parameters are underlined).

Sample	Path	*N*	*R* (Å)	σ^2 (10^{-3}Å^2)	ΔE_0 (eV)	*R*-factor
Ex-situ	Ru-N	4.2±0.3	2.08±0.02	2.4±1.4	2.3±1.3	0.002
At open circuit	Ru-N	4.2±0.3	2.08±0.02	2.4±1.4	2.3±1.3	0.002
At 1.5V	Ru-N	<u>4.2</u>	2.06±0.02	3.1±1.5	<u>2.3</u>	0.003
	Ru-O	1.0±0.2	2.00±0.02	3.0±1.5	3.0±1.5	

N, coordination number; *R*, bonding distance; σ^2 Debye-Waller factor; ΔE_0 inner potential shift.

Supplementary Table 2. DFT calculations for vibrational absorption band in several possible configurations. The red bold represent the vibrated functional groups.

Surface-adsorbed species	Wavenumber (cm^{-1})
**O**-(Ru_1-N_4)	782.3
**O**-(Ru_1-N_4)-OH	802.8
**O**-(Ru_1-N_4)-O	783.9
**O**-(Ru_1-N_4)-OOH	790.4
O-(Ru_1-N_4)-**O**	838.0
O-(Ru_1-N_4)-**OH**	917.7
O-(Ru_1-N_4)-**OOH**	1327.8

Supplementary Table 3. The comparisons of OER activity of the Ru-N-C with other recently reported catalysts in acid media.

Catalyst	Mass loading (mg/cm^2)	η(mV)	Mass activity ($A\ g_{metal}^{-1}$)	Electrolyte	References
Ru-N-C	0.28	267	3540(@267 mV)	0.5 M H_2SO_4	This work
Ru	N/A	340	N/A	0.1 M H_2SO_4	Ref.10
r-RuO_2 Nps	0.05	> 420	11(@250 mV)	0.1 M $HClO_4$	Ref.11
r-IrO_2 Nps	0.05	> 420	3.5(@250 mV)	0.1 M $HClO_4$	Ref.11
Ir/$SrIrO_3$	N/A	270	N/A	0.5 M H_2SO_4	Ref.12
Ru_1-Pt_3Cu	0.0163_{Pt+Ru}	220	779(@250 mV)	0.1 M $HClO_4$	Ref.13
Ba[Co-POM]	N/A	361	N/A	1.0 M H_2SO_4	Ref.14
Ir	0.82	290	N/A	0.5 M H_2SO_4	Ref.15
Ir/Au	N/A	410	N/A	0.1 M H_2SO_4	Ref.16
IrNiCu DNF	0.16	303±4	460 (@300 mV)	0.1 M $HClO_4$	Ref.17
IrO_x/ATO	0.0102	360	39.1(@280 mV)	0.05 M H_2SO_4	Ref.18
$Y_2Ru_2O_{7-\delta}$	2.9	270(@2.23 mA cm^{-2})	N/A	0.1 M $HClO_4$	Ref.19
$Y_2[Ru_{1.6}Y_{0.4}]O_{7-\delta}$	~0.06	~250	~600(@270 mV)	0.1 M $HClO_4$	Ref.20
$IrNiO_x$/ Meso-ATO	10.2	~320	~90(@280 mV)	0.05 M H_2SO_4	Ref.21
$Cr_{0.6}Ru_{0.4}O_2$ (500)	0.28	178	229(@270 mV)	0.05 M H_2SO_4	Ref.22
IrO_2-RuO_2@Ru	0.379	281	N/A	0.5 M H_2SO_4	Ref.23
IrOx-Ir	130 $\mu g_{Ir}\ cm^{-2}$	290	N/A	0.5 M H_2SO_4	Ref.24
$NaRuO_2$	0.2	~255	42(@250 mV)	0.1 M $HClO_4$	Ref.25

The overpotential (η) were recorded at the current density of 10 mA cm^{-2}.

Supplementary Table 4. The Free energy diagram of OER on Ru_1-N_4 (Red line), O-Ru_1-N_4 (blue line) and HO-Ru_1-N_4 (green line).

Sample	Adsorbate	ZPE (eV)	TS (eV)
H_2O	/	0.59	0.67
H_2	/	0.27	0.41
Ru_1-N_4	OH^*	0.36	0.09
	O*	0.07	0.07
	OOH*	0.43	0.20
O-Ru_1-N_4	OH^*	0.37	0.08
	O*	0.08	0.05
	OOH*	0.45	0.19
HO-Ru_1-N_4	OH^*	0.36	0.09
	O*	0.07	0.06
	OOH*	0.44	0.16

Supplementary References

1. Nørskov, J. K. *et al.* Origin of the Overpotential for Oxygen Reduction at a Fuel-Cell Cathode. *J. Phys. Chem. B* **108**, 17886-17892 (2004).
2. Wang, X. *et al.* A metal-free polymeric photocatalyst for hydrogen production from water under visible light. *Nat. Mater.* **8**, 76 (2009).
3. Chen, I. L. *et al.* Local structure distortion induced by Ti dopants boosting the pseudocapacitance of RuO_2-based supercapacitors. *Nanoscale* **7**, 15450-15461 (2015).
4. Liu, J. *et al.* Metal-free efficient photocatalyst for stable visible water splitting via a two-electron pathway. *Science* **347**, 970-974 (2015).
5. Chen, I.-L. *et al.* Local structure distortion induced by Ti dopants boosting the pseudocapacitance of RuO 2-based supercapacitors. *Nanoscale* **7**, 15450-15461 (2015).
6. Ananth, A., Gandhi, M. S. & Mok, Y. S. A dielectric barrier discharge (DBD) plasma reactor: an efficient tool to prepare novel RuO_2 nanorods. *J. Phys. D: Appl. Phys.* **46**, 155202 (2013).
7. Ran, J., Ma, T. Y., Gao, G., Du, X.-W. & Qiao, S. Z. Porous P-doped graphitic carbon nitride nanosheets for synergistically enhanced visible-light photocatalytic H_2 production. *Energy Environ.Sci.* **8**, 3708-3717 (2015).
8. Zhang, Y., Mori, T., Ye, J. & Antonietti, M. Phosphorus-doped carbon nitride solid: enhanced electrical conductivity and photocurrent generation. *J. Am. Chem. Soc.* **132**, 6294-6295 (2010).
9. Cao, L. *et al.* Identification of single-atom active sites in carbon-based cobalt catalysts during electrocatalytic hydrogen evolution. *Nat. Catal.* **2**, 134 (2019).
10. McCrory, C. C., Jung, S., Peters, J. C. & Jaramillo, T. F. Benchmarking heterogeneous electrocatalysts for the oxygen evolution reaction. *J. Am. Chem. Soc.* **135**, 16977-16987 (2013).
11. Lee, Y., Suntivich, J., May, K. J., Perry, E. E. & Shao-Horn, Y. Synthesis and activities of rutile IrO_2 and RuO_2 nanoparticles for oxygen evolution in acid and alkaline solutions. *J. Phys. Chem. Lett.* **3**, 399-404 (2012).
12. Seitz, L. C. *et al.* A highly active and stable $IrO_x/SrIrO_3$ catalyst for the oxygen evolution reaction. *Science* **353**, 1011-1014 (2016).
13. Yao, Y. *et al.* Engineering the electronic structure of single atom Ru sites via compressive strain boosts acidic water oxidation electrocatalysis. *Nat. Catal. 2, 304-313* (2019).
14. Blasco-Ahicart, M., Soriano-López, J., Carbó, J. J., Poblet, J. M. & Galan-Mascaros, J. Polyoxometalate electrocatalysts based on earth-abundant metals for efficient water oxidation in acidic media. *Nat. Chem.* **10**, 24 (2018).
15. Zhang, J. *et al.* Iridium nanoparticles anchored on 3D graphite foam as a bifunctional electrocatalyst for excellent overall water splitting in acidic solution. *Nano energy* **40**, 27-33 (2017).
16. Ahn, S. H. *et al.* Self-terminated electrodeposition of iridium electrocatalysts. *Energy Environ.Sci.* **8**, 3557-3562 (2015).
17. Park, J. *et al.* Iridium-based multimetallic nanoframe@ nanoframe structure: an efficient and robust electrocatalyst toward oxygen evolution reaction. *ACS nano* **11**, 5500-5509 (2017).
18. Oh, H.-S. *et al.* Electrochemical catalyst–support effects and their stabilizing role for IrO_x nanoparticle catalysts during the oxygen evolution reaction. *J. Am. Chem. Soc.* **138**, 12552-12563 (2016).
19. Kim, J. *et al.* High-performance pyrochlore-type yttrium ruthenate electrocatalyst for oxygen

evolution reaction in acidic media. *J. Am. Chem. Soc.* **139**, 12076-12083 (2017).

20. Kim, J. *et al.* A Porous Pyrochlore Y_2 $[Ru_{1.6}Y_{0.4}]$ $O_{7-\delta}$ Electrocatalyst for Enhanced Performance towards the Oxygen Evolution Reaction in Acidic Media. *Angew. Chem. Int. Ed.* **130**, 14073-14077 (2018).
21. Nong, H. N. *et al.* Oxide-Supported $IrNiO_x$ Core-Shell Particles as Efficient, Cost-Effective, and Stable Catalysts for Electrochemical Water Splitting. *Angew. Chem. Int. Ed.* **54**, 2975-2979 (2015).
22. Lin, Y. *et al.* Chromium-ruthenium oxide solid solution electrocatalyst for highly efficient oxygen evolution reaction in acidic media. *Nat. Commun.* **10**, 162 (2019).
23. Li, G., Li, S., Ge, J., Liu, C. & Xing, W. Discontinuously covered IrO_2-RuO_2@ Ru electrocatalysts for the oxygen evolution reaction: how high activity and long-term durability can be simultaneously realized in the synergistic and hybrid nano-structure. *J.Mater.Chem.A* **5**, 17221-17229 (2017).
24. Lettenmeier, P. *et al.* Nanosized IrO_x-Ir Catalyst with Relevant Activity for Anodes of Proton Exchange Membrane Electrolysis Produced by a Cost-Effective Procedure. *Angew. Chem. Int. Ed.* **128**, 752-756 (2016).
25. Laha, S. *et al.* Ruthenium Oxide Nanosheets for Enhanced Oxygen Evolution Catalysis in Acidic Medium. *Adv.Energy.Mater.* **9**, 1803795 (2019).
